# Aneurysmal Bone Cyst of the Distal Femoral Metaphysis in a Four-year-old Female Patient Presenting with a Pathologic Fracture: A Case Report

**DOI:** 10.7759/cureus.4846

**Published:** 2019-06-06

**Authors:** James Dorosh, Parth Vyas

**Affiliations:** 1 Orthopedic Oncology, Sanford Medical Center, Fargo, USA

**Keywords:** aneurysmal bone cyst, telangiectatic osteosarcoma, orthopedic oncology, capanna criteria, bone curettage, pathological fracture

## Abstract

Aneurysmal bone cysts (ABCs) are benign osteolytic vascular lesions that are capable of aggressive local expansion and bone destruction. These tumors are most common in adolescent patients and constitute approximately 9% of benign tumors. ABCs can present a diagnostic challenge, as they share several histological and radiographic characteristics with more aggressive lesions, including giant cell tumors and malignant telangiectatic osteosarcomas. The management of ABCs is diverse, but the most common approach includes lesion curettage with bone grafting. Here, we present the case of a large, central ABC of the distal femur in a young, previously healthy female who presented to the emergency room with a pathologic fracture.

## Introduction

Aneurysmal bone cysts (ABCs) are benign osteolytic vascular lesions that are capable of aggressive local expansion and bone destruction [1]. Because of this, morbidity and symptomatology tend to arise from local tissue destruction and pathologic fracture. Patients typically present during the second decade of life and ABCs represent approximately 9% of benign bone tumors [2]. Care must be taken to differentiate the diagnosis of ABC from a unicameral bone cyst and telangiectatic osteosarcoma as the treatment protocol is different [3]. Tissue biopsy with histologic interpretation must be consistently employed to avoid misdiagnosis. If the lesion is amenable, curettage with bone grafting is the recommended initial approach to management [4]. Adjuvant therapies are multiple and include electrocautery, cryotherapy, intra-lesional sclerotherapy, preoperative embolization, and medical therapy with denosumab as fracture prophylaxis [4-5]. Despite appropriate management, reports of tumor recurrence range considerably, with different treatment options from as low as 5% to more than 40% [6].

Here, we will discuss the unique case of a large central Type 1 Campanacci criteria ABC of the distal femur in a young, previously healthy female who presented to the emergency room with a pathologic fracture after sustaining a fall from the standing height. This paper will also review the different radiographic types of ABCs, discuss the radiographic and histologic differentiation from telangiectatic osteosarcoma, and discuss the recommended management.

## Case presentation

A four-year-old girl presented to the emergency department (ED) complaining of sudden onset severe left thigh pain after sustaining a fall from the standing height while playing a game of tag. The patient’s parents denied pain with ambulation prior to this episode, previous nocturnal pain, a family or patient history of cancer, fever, chills, weight loss, or any other symptoms. On physical exam, the patient was in moderate distress and screamed at any attempt to move her leg. She was unable to bear weight on the affected leg and the thigh was exquisitely tender to palpation with diffuse swelling.

**Figure 1:**
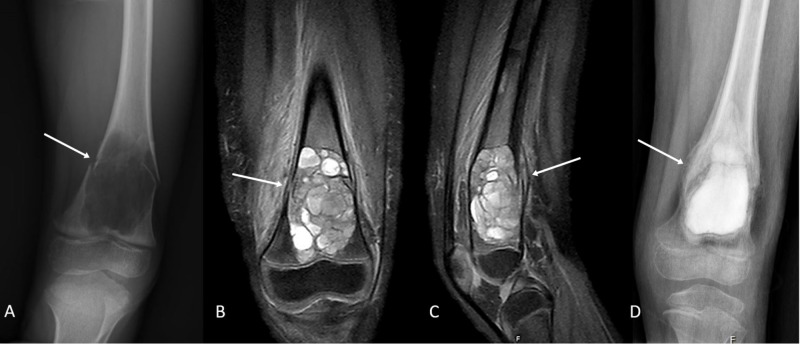
(A) Plain anteroposterior radiographic film demonstrating a left femoral distal metaphyseal expansile lytic lesion with a fallen leaf type fracture. (B,C) Coronal and sagittal magnetic resonance (MR) images demonstrating expansile lesion of the distal metaphysis of the femur. No underlying soft tissue elements are noted, suggesting the lesion is most likely a primary aneurysmal bone cyst. (D) Plain anteroposterior radiographic film demonstrating callus formation and postop changes at six weeks of follow-up.

X-rays demonstrated an expansile lytic lesion of the left distal femur with a pathologic fracture and a positive fallen leaf sign (Figure 1A). Magnetic resonance (MR) imaging showed a lesion measuring 3.5 x 3.0 cm in the greatest axial cross-section and 5.2 cm in the craniocaudal extent. The lesion was centered within the metaphysis and appeared expansile with surrounding cortical thinning, without invasion of the physis. There were multiple blood-filled cystic spaces with fluid levels. There were no underlying soft tissue elements noted, suggesting this was a primary ABC (Figures 1B-1C).

**Figure 2:**
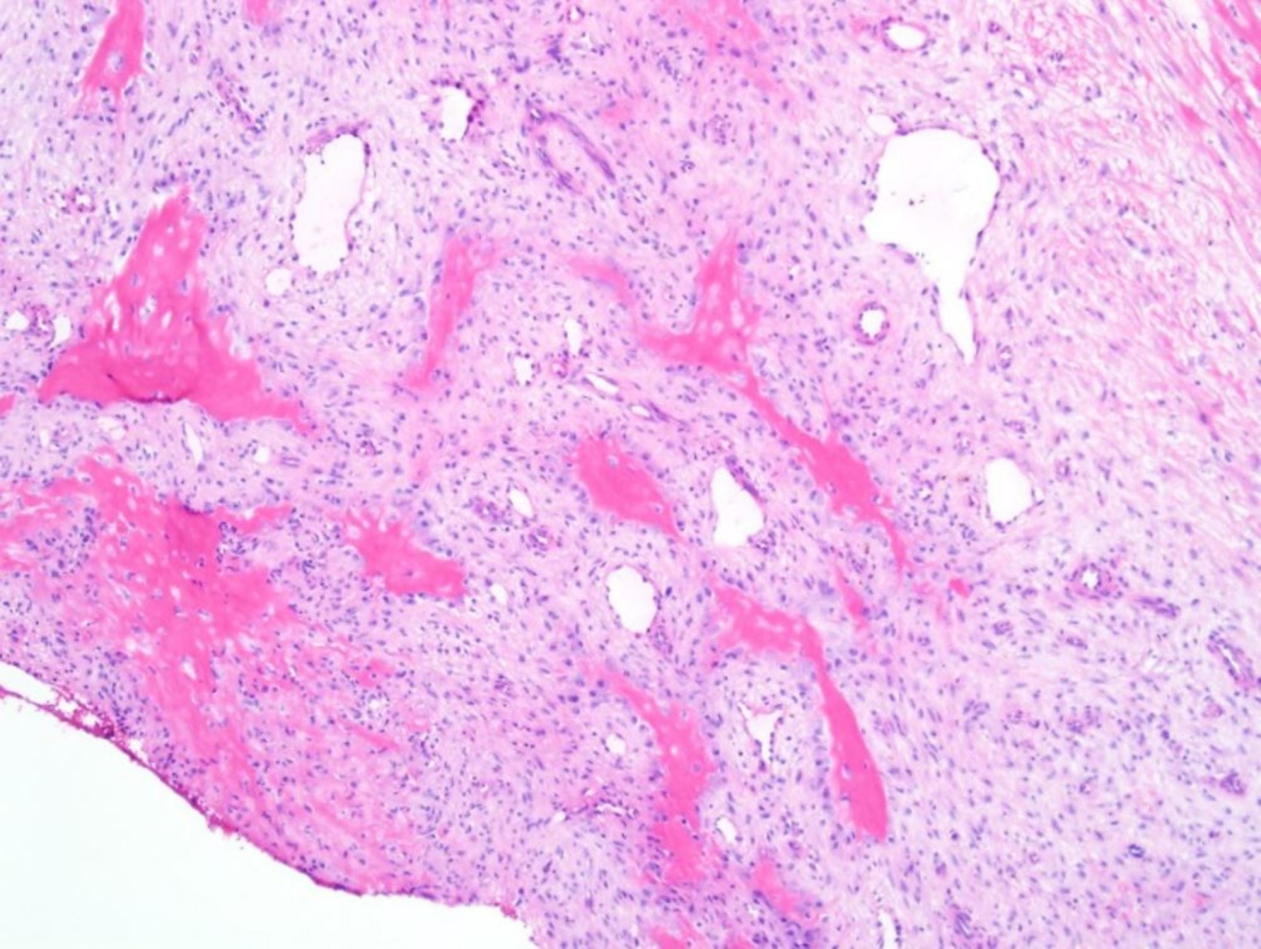
Stained histologic section demonstrating a cystic lesion composed of fibroblasts and myofibroblasts admixed with seams of osteoid and scattered multinucleated giant cells without significant cellular atypia consistent with an aneurysmal bone cyst.

Left femoral bone cyst curettage and bone grafting were performed. First, the femur was exposed through a 3 cm lateral incision of the distal thigh and a periosteal window in the bone was created. Electrocautery was used to achieve hemostasis and lesion curettage was performed. Tissue samples were analyzed as a frozen section and, once determined to be a benign lesion, the lesion was thoroughly curetted. Chemical adjuvant was withheld secondary to the close proximity of the lesion to the growth plate. A titanium nail was used to establish communication between the various cystic components of the lesion and the bony medullary canal. A bone void filler consisting of calcium sulfate and calcium phosphate was placed under X-ray guidance. After confirming appropriate fracture reduction, the patient was closed and placed in a Spica cast. The formal pathological report demonstrated a cystic lesion with bland fibroblasts and myofibroblasts admixed with seams of osteoid and scattered multinucleated giant cells without significant cellular atypia suggestive of ABC (Figure 2).

At the six-week follow-up appointment, X-rays demonstrated interval consolidation of the bone graft with callus formation at the fracture site (Figure 1D). There was no sign of lesion recurrence and the patient was able to return to full preoperative weight-bearing activities.

## Discussion

The incidence of ABCs is approximately 0.14 per 100,000 persons each year, and they typically present during a patient’s adolescent years [3]. This case represents a minority of patients that can present at an earlier date. The skeletal location of distal femoral metaphysis is an important weight-bearing location where the patient's first presentation is commonly following trauma and fracture. This presentation can complicate treatment.

In 1986, a radiographic classification system of ABCs, along with the recommended management and expected outcomes, was presented by Campanacci et al. These ABC grading criteria are often referred to in an abbreviated manner simply as the “Campanacci criteria,” as shown in Table 1. These criteria distinguish amongst five unique ABC types based on their radiographic appearance. Briefly, Type 1 cysts are centrally arising and can spread to the cortical edge of bones, sometimes enlarging the total diameter of the bone. They are typically located in the metaphysis or metadiaphysis of long bones. Type 2 cysts are centrally located and replace the entirety of the involved boney segment within which they occur. Type 3 cysts arise eccentrically, with little to no cortical involvement. Type 4 cysts are subperiosteal, usually arising at the diaphysis of long bones, and lift the periosteum circumferentially as they expand. Finally, Type 5 cysts are also subperiosteal and elevate the periosteum circumferentially, as in Type 4; however, they also destroy the adjacent cortical and cancellous bone as they enlarge [7].

**Table 1:** Abbreviated Campanacci classification criteria of aneurysmal bone cyst types

Type	Osteologic Site	Skeletal Site	Radiographic Appearance
1	central	long bone metaphysis and metadiaphysis	cystic lucency with cortical attenuation
2	central expansive	long bone metaphysis and metadiaphysis	cystic lucency with severe cortical distension
3	eccentric	long bone metaphysis	variable lucency
4	subperiosteal	long bone diaphysis	periosteal elevation
5	subperiosteal expansive	cancellous bone	periosteal elevation with cortical destruction

Per the Campanacci criteria, our case exemplifies a Type 1 ABC. This type of ABC makes up approximately 23% of ABCs and curettage through a boney window, as completed in our case, is the recommended treatment [7]. Today, the Campanacci criteria remain a useful method of categorizing ABC subtypes and their respective treatments.

Making the correct diagnosis is vitally important for the management of an ABC. The most important alternative diagnosis that one must consider is telangiectatic osteosarcoma (TOC). TOCs are malignant primary bone neoplasms that are highly vascularized, lacking the expected solid osteoid production characteristic of typical osteosarcomas [8]. TOC presents as multiple cystic compartments on imaging, with fluid levels making it easy to confuse with an ABC [9]. The age of presentation between these two conditions is also similar, further complicating differentiation. Careful diagnosis is vital, as the treatment of TOC is based on that of osteosarcomas [10].

Radiographically, TOCs tend to grow more rapidly and invade nearby structures more consistently than ABCs. producing a soft tissue mass [11-12]. However, these basic radiographic tendencies can be insufficient for reliable diagnosis via imaging alone. Therefore, a biopsy is performed for further differentiation. The primary means of differentiating these two tumors histologically is the presence of cellular atypia. TOC is characterized by “highly atypical stromal cells with hyperchromasia, nuclear pleomorphism, and atypical mitoses" [11]. ABCs lack these malignant changes, making their differentiation possible under the microscope.

There are a variety of approaches to the management of ABCs, ranging from medical therapy alone to curettage with grafting and even wide resection. Each method, with its respective risks and outcomes, presents patients and providers with the question of effective tumor management vs total morbidity and mortality. As detailed by Schreuder et al., wide resection vs curettage and bone grafting was associated with a recurrence rate of 0% vs approximately 31%, respectively; however, wide resection was associated with a greater need for reconstructive surgery and its associated increased morbidity [13]. Because of this, it is reasonable to accept the relatively higher tumor recurrence rate associated with curettage and bone grafting alone as compared to wide resection based on the goal of a more rapid return to a functional baseline as demonstrated in our clinical case.

## Conclusions

ABCs can present a diagnostic challenge even to the most skilled practitioner. Lesions of the distal femur can present as pathologic fractures and must be differentiated from the more ominous TOC. Once a diagnosis is established, the Campanacci criteria remain a useful starting point for lesion categorization and basic management. Treatment options are many, but curettage with bone grafting provides an acceptable compromise between tumor-free survival and treatment morbidity and mortality.
